# Bis­(di­meth­oxy­ethane-1κ^2^ *O*,*O*′)penta­kis­(1,1,1,3,3,3-hexa­fluoro­propan-2-olato)-2κ^3^ *O*,3κ^2^ *O*-μ-hy­droxido-1:3κ^2^ *O*-μ_3_-oxido-1:2:3κ^3^ *O*-magnesiumdialuminium

**DOI:** 10.1107/S2414314623007162

**Published:** 2023-09-08

**Authors:** Matic Lozinšek, Tjaša Pavčnik, Jan Bitenc

**Affiliations:** a Jožef Stefan Institute, Jamova cesta 39, 1000 Ljubljana, Slovenia; b National Institute of Chemistry, Hajdrihova 19, 1000 Ljubljana, Slovenia; c Faculty of Chemistry and Chemical Technology, University of Ljubljana, Večna pot 113, 1000 Ljubljana, Slovenia; University of Aberdeen, United Kingdom

**Keywords:** fluorinated alk­oxy­aluminate, hfip, magnesium battery electrolyte, crystal structure

## Abstract

Partial hydrolysis of a sample of [Mg(dme)_3_][Al(hfip)_4_]_2_ crystals led to the formation of the title complex, [Mg(dme)_2_{HOAl(hfip)_2_OAl(hfip)_3_}] (dme = di­meth­oxy­ethane and hfipH = hexa­fluoro­iso­propanol) or [Mg(C_4_H_10_O_2_)_2_O(OH)Al_2_(C_3_HF_6_O)_5_]. The magnesium cation exhibits a distorted octa­hedral coordination with two bidentate di­meth­oxy­ethane mol­ecules and a dinuclear aluminate anion, coordinated to Mg^2+^ *via* oxido and hydroxido units. The anion is an oxido-bridged species, [HOAl(hfip)_2_(μ-O)Al(hfip)_3_]^2–^, with one Al^3+^ cation tetra­hedrally coordinated by an oxido (O^2–^) anion, a hydroxido anion, and two hfip groups, whereas the second Al^3+^ cation is coordinated by the oxido anion and three hfip groups.

## Abstract 2

The title complex was formed as a partial hydrolysis product of [Mg(dme)_3_][Al(hfip)_4_]_2_ (dme = di­meth­oxy­ethane and hfipH = hexa­fluoro­iso­propanol), a promising electrolyte salt for magnesium batteries.

## Graphical abstract

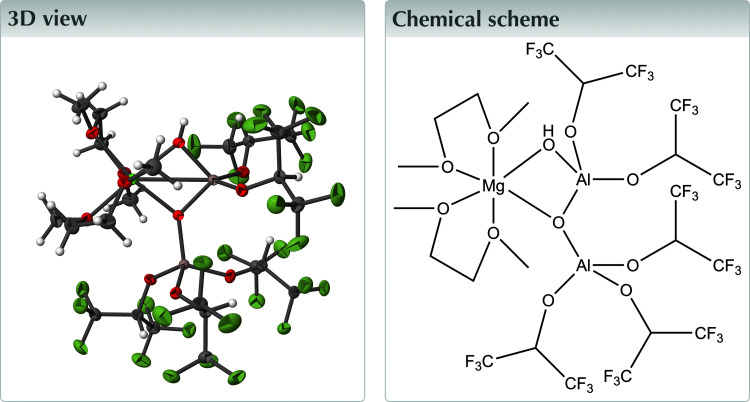

## Structure description

Salts of weakly coordinating anions (Barthélemy *et al.*, 2023 ▸), such as [Al(hfip)_4_]^−^ [tetra­kis­(1,1,1,3,3,3-hexa­fluoro­propan-2-olato)aluminate; hfipH = hexa­fluoro­iso­pro­pan­ol], have recently emerged as state-of-the-art electrolytes for rechargeable multivalent metal batteries (Herb *et al.*, 2016 ▸; Mandai *et al.*, 2021 ▸; Pavčnik *et al.*, 2023 ▸). The title compound formed upon partial hydrolysis of the complex [Mg(dme)_3_][Al(hfip)_4_]_2_ (dme = di­meth­oxy­ethane), which is a promising electrolyte salt for magnesium batteries (Pavčnik *et al.*, 2022 ▸).

**Figure 1:**
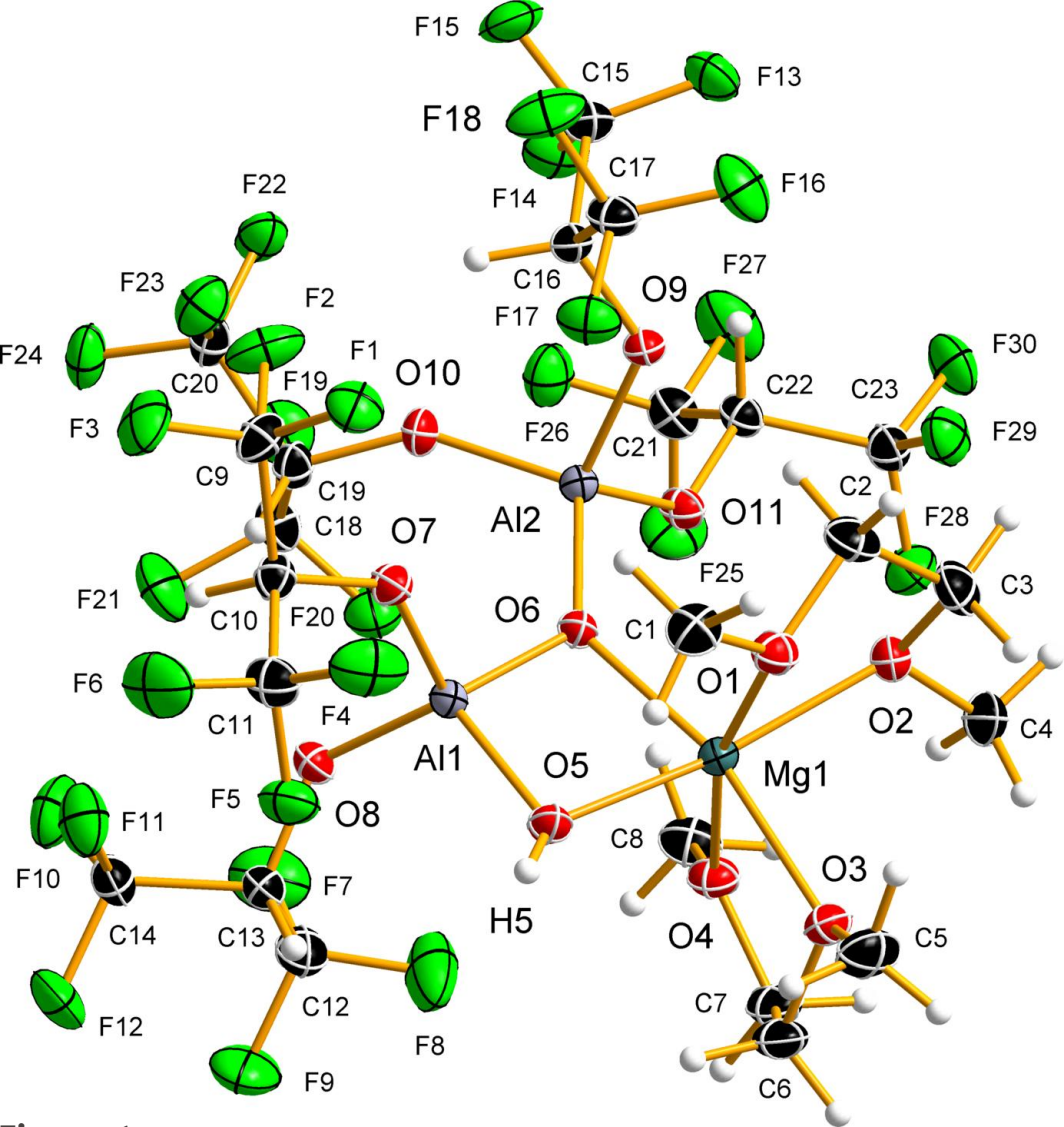
The asymmetric unit and selected atom labels of the [Mg(dme)_2_{HOAl(hfip)_2_OAl(hfip)_3_}] crystal structure (dme: di­meth­oxy­ethane, hfip: 1,1,1,3,3,3-hexa­fluoro­iso­propano­lato). Displacement ellipsoids are plot­ted at the 50% probability level and hydrogen atoms are depicted as small spheres of arbitrary radius.

The title compound crystallizes in the monoclinic space group *P*2_1_/*n* with four mol­ecules in the unit cell. The magnesium cation is coordinated by two bidentate dme mol­ecules with Mg—O distances of 2.0813 (6)–2.1185 (6) Å and by the oxido and hydroxido groups of the anion with slightly shorter Mg—O bond lengths of 2.0383 (6) and 2.0470 (6) Å, respectively (Fig. 1 ▸). In the dinuclear [HOAl(hfip)_2_OAl(hfip)_3_]^2–^ anion, the first central aluminium cation, Al1, is coordinated by two hfip ((CF_3_)_2_CHO–) [1.7374 (6), 1.7425 (6) Å], hydroxido [1.7644 (6) Å] and a bridging oxido ligand [1.7456 (6) Å], whereas the second Al^3+^ cation, Al2, is coordinated by three hfip moieties [1.7307 (6)–1.7645 (6) Å] and by the oxido bridge [1.7384 (6) Å] (Fig. 1 ▸). The tetra­hedral shape of the [AlO_4_] unit is more distorted in the case of the Al1 atom than in the case of the Al2 atom, with the corresponding O—Al—O angles being 92.84 (3)–117.93 (3)° and 102.33 (3)–115.11 (3)°, respectively. The nearly right angle involves the oxido and hydroxido groups (O—Al1—OH). The anion coordinates to the magnesium cation *via* hydroxido and oxido units, thus making these ligands μ- and μ_3_-bridges, respectively, resulting in an Mg1⋯Al1 distance of 2.8074 (3) Å. The angles at the hydroxido and oxido bridges between Al1 and Mg1 are similar [Al1—O5—Mg1 = 94.59 (3)°, Al1—O6—Mg1 = 95.48 (3)°], whereas the angles involving μ_3_-oxido and Al2 are more obtuse [Al1—O6—Al2 = 128.72 (3)°, Al2—O6—Mg1 = 135.07 (3)°]. The Mg^2+^ cation has a distorted octa­hedral coordination with *cis-*O—Mg—O angles ranging from 76.40° (2) to 101.00 (2)° and *trans-*O—Mg—O angles in the range 164.77 (3)–168.38 (3)°. The O—Mg—O bite angles of the dme ligands [76.40 (3) and 77.08 (3)°] and the anion [76.99 (2)°] are nearly identical.

Similar Mg—O distances and bite angles have been observed in other magnesium complexes with coordinating dme, for example: 2.0688 (11), 2.1146 (12) Å, 77.49 (5)° in [Mg(dme)_2_(CF_3_SO_3_)_2_] (Cambridge Structural Database refcode EJUYEQ; Nguyen *et al.*, 2020 ▸) and 2.0645 (12)–2.0854 (13) Å, 77.39 (5)–78.25 (5)° in [Mg(dme)_3_](CB_9_H_10_)_2_ (LATRUW; McArthur *et al.*, 2017 ▸). The Al—O(hfip) distances of the title compound are in agreement with bond lengths observed in compounds of the [Al(hfip)_4_]^−^ anion: 1.7367 (10)–1.7444 (10) Å in NMe_4_[Al(hfip)_4_] (FOZRIW; Raabe *et al.*, 2009 ▸) and 1.7140 (13)–1.7624 (14) Å in [Ag(CO)_2_Al(hfip)_4_] (XARFED; Schaefer *et al.*, 2013 ▸). The geometric parameters of the CF_3_ groups are consistent with those reported in crystal structures of other tri­fluoro­methyl­ated organic compounds (Motaln *et al.*, 2023 ▸; Radan *et al.*, 2023 ▸).

In the extended structure, the hydroxido unit forms an inter­molecular O—H⋯F hydrogen bond (Table 1 ▸) to the hfip moiety of the OAl(hfip)_3_ group.

Bond-valence calculations (Brown, 2009 ▸, 2016 ▸) for the magnesium, aluminium, and oxygen atoms of the μ-hydroxido and μ_3_-oxido ligands agree well with the expected values (in valence units) and confirm the atom assignments: Mg1 2.09, Al1 3.08, Al2 3.10, O5 1.95, O6 1.96. Calculations were performed using the following parameters: *b* = 0.37 Å, *R*_0_ = 1.693 Å (Mg—O), 1.651 Å (Al—O); and *b* = 0.94 Å, *R*_0_ = 0.569 Å (H—O) (Brown & Altermatt, 1985 ▸; Brown, 2020 ▸, 2016 ▸).

## Synthesis and crystallization

Single crystals of the title compound formed in a partial hydrolysis of the [Mg(dme)_3_][Al(hfip)_4_] sample (Pavčnik *et al.*, 2022 ▸) that was kept on a watch glass under the layer of perfluoro­deca­line, upon storage in refrigerator at about 8 °C for a day. The formation of this hydrolysis product could be tentatively described with the following equation:

[Mg(dme)_3_][Al(hfip)_4_]_2_ + 2H_2_O → [Mg(dme)_2_{HOAl(hfip)_2_OAl(hfip)_3_}] + dme + 3hfipH.

## Refinement

The crystal data, data collection, and structure refinement details are summarized in Table 2 ▸. Hydrogen atoms were refined freely including their isotropic thermal parameters (Cooper *et al.*, 2010 ▸).

## Supplementary Material

Crystal structure: contains datablock(s) I. DOI: 10.1107/S2414314623007162/hb4443sup1.cif

Structure factors: contains datablock(s) I. DOI: 10.1107/S2414314623007162/hb4443Isup2.hkl

CCDC reference: 2156683

Additional supporting information:  crystallographic information; 3D view; checkCIF report

## Figures and Tables

**Table 1:** Hydrogen-bond geometry (Å, °)

*D*—H⋯*A*	*D*—H	H⋯*A*	*D*⋯*A*	*D*—H⋯*A*
O5—H5⋯F18^i^	0.750 (18)	2.315 (18)	2.9816 (8)	148.7 (17)

Symmetry code: (i) 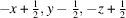 .

**Table 2:** Experimental details

Crystal data	
Chemical formula	[Mg(C_4_H_10_O_2_)_2_O(OH)Al_2_(C_3_HF_6_O)_5_]
*M*_r_	1126.71
Crystal system, space group	Monoclinic, *P*2_1_/*n*
Temperature (K)	100
*a*, *b*, *c* (Å)	10.68706 (9), 19.53919 (16), 19.31983 (17)
β (°)	91.7888 (7)
*V* (Å^3^)	4032.33 (6)
*Z*	4
Radiation type	Ag *K*α, λ = 0.56087 Å
μ (mm^−1^)	0.15
Crystal size (mm)	0.74 × 0.58 × 0.36

Data collection	
Diffractometer	XtaLAB Synergy-S, Dualflex, Eiger2 R CdTe 1M
Absorption correction	Gaussian (*CrysAlis PRO*; Rigaku OD, 2022 ▸)
*T*_min_, *T*_max_	0.190, 1.000
No. of measured, independent and observed [*I* > 2σ(*I*)] reflections	271135, 20244, 16165
*R*_int_	0.030
(sin θ/λ)_max_ (Å^−1^)	0.870

Refinement	
*R*[*F* ^2^ > 2σ(*F* ^2^)], *wR*(*F* ^2^), *S*	0.033, 0.091, 1.04
No. of reflections	20244
No. of parameters	708
H-atom treatment	All H-atom parameters refined
Δρ_max_, Δρ_min_ (e Å^−3^)	0.51, −0.41

Computer programs: *CrysAlis PRO* (Rigaku OD, 2022 ▸), *OLEX2.solve* (Dolomanov *et al.*, 2009 ▸), *OLEX2* (Dolomanov *et al.*, 2009 ▸), *SHELXL2019/2* (Sheldrick, 2015 ▸), *DIAMOND* (Brandenburg, 2005 ▸) and *publCIF* (Westrip, 2010 ▸).

## full crystallographic data

### Crystal data

**Table:**

[Al_2_Mg(C_3_HF_6_O)_5_O(OH)(C_4_H_10_O_2_)_2_]	*F*(000) = 2240
*M**_r_* = 1126.71	*D*_x_ = 1.856 Mg m^−^^3^
Monoclinic, *P*2_1_/*n*	Ag *K*α radiation, λ = 0.56087 Å
*a* = 10.68706 (9) Å	Cell parameters from 111325 reflections
*b* = 19.53919 (16) Å	θ = 1.9–29.3°
*c* = 19.31983 (17) Å	µ = 0.15 mm^−^^1^
β = 91.7888 (7)°	*T* = 100 K
*V* = 4032.33 (6) Å^3^	Block, colourless
*Z* = 4	0.74 × 0.58 × 0.36 mm

### Data collection

**Table:**

XtaLAB Synergy, Dualflex, Eiger2 R CdTe 1M diffractometer	20244 independent reflections
Radiation source: micro-focus sealed X-ray tube, PhotonJet (Ag) X-ray Source	16165 reflections with *I* > 2σ(*I*)
Mirror monochromator	*R*_int_ = 0.030
Detector resolution: 13.3333 pixels mm^-1^	θ_max_ = 29.2°, θ_min_ = 1.7°
ω scans	*h* = −18→17
Absorption correction: gaussian (CrysAlisPro; Rigaku OD, 2022)	*k* = −31→29
*T*_min_ = 0.190, *T*_max_ = 1.000	*l* = −33→31
271135 measured reflections	

### Refinement

**Table:**

Refinement on *F*^2^	Primary atom site location: iterative
Least-squares matrix: full	Hydrogen site location: difference Fourier map
*R*[*F*^2^ > 2σ(*F*^2^)] = 0.033	All H-atom parameters refined
*wR*(*F*^2^) = 0.091	*w* = 1/[σ^2^(*F*_o_^2^) + (0.0393*P*)^2^ + 1.1133*P*] where *P* = (*F*_o_^2^ + 2*F*_c_^2^)/3
*S* = 1.04	(Δ/σ)_max_ = 0.001
20244 reflections	Δρ_max_ = 0.51 e Å^−^^3^
708 parameters	Δρ_min_ = −0.41 e Å^−^^3^
0 restraints	

### Special details

**Table:**

Geometry. All esds (except the esd in the dihedral angle between two l.s. planes) are estimated using the full covariance matrix. The cell esds are taken into account individually in the estimation of esds in distances, angles and torsion angles; correlations between esds in cell parameters are only used when they are defined by crystal symmetry. An approximate (isotropic) treatment of cell esds is used for estimating esds involving l.s. planes.

### Fractional atomic coordinates and isotropic or equivalent isotropic displacement parameters (Å^2^)

**Table:**

	*x*	*y*	*z*	*U*_iso_*/*U*_eq_	
Al1	0.41472 (2)	0.33992 (2)	0.19998 (2)	0.01624 (4)	
Al2	0.56934 (2)	0.45794 (2)	0.27249 (2)	0.01543 (4)	
Mg1	0.46648 (3)	0.30191 (2)	0.33805 (2)	0.01728 (5)	
F1	0.06620 (6)	0.46975 (3)	0.17179 (3)	0.03163 (12)	
F2	0.22151 (6)	0.51573 (3)	0.12258 (4)	0.04067 (16)	
F3	0.07180 (7)	0.47128 (4)	0.06062 (4)	0.04243 (16)	
F4	0.04732 (7)	0.33194 (4)	0.16943 (5)	0.04363 (17)	
F5	0.19142 (6)	0.27618 (3)	0.11852 (4)	0.03338 (12)	
F6	0.05329 (8)	0.33255 (4)	0.05821 (5)	0.0517 (2)	
F7	0.73093 (7)	0.27815 (4)	0.09021 (5)	0.04776 (18)	
F8	0.66025 (9)	0.21707 (5)	0.17359 (4)	0.0572 (2)	
F9	0.67081 (8)	0.17495 (4)	0.07174 (4)	0.04579 (17)	
F10	0.55645 (7)	0.30952 (4)	−0.01500 (3)	0.04094 (15)	
F11	0.36460 (6)	0.28918 (4)	0.00501 (3)	0.03925 (14)	
F12	0.48778 (10)	0.20632 (4)	−0.01903 (4)	0.0544 (2)	
F13	0.49310 (7)	0.65885 (3)	0.36062 (3)	0.03434 (13)	
F14	0.60216 (6)	0.63480 (3)	0.27238 (4)	0.03464 (13)	
F15	0.43057 (7)	0.69180 (3)	0.25870 (4)	0.03847 (14)	
F16	0.27865 (6)	0.58285 (4)	0.37760 (3)	0.03851 (14)	
F17	0.22303 (5)	0.51681 (3)	0.29389 (3)	0.03107 (12)	
F18	0.21428 (6)	0.62570 (3)	0.27978 (5)	0.04278 (17)	
F19	0.81725 (6)	0.51700 (4)	0.11235 (4)	0.03499 (13)	
F20	0.77974 (6)	0.41675 (3)	0.15412 (4)	0.03677 (13)	
F21	0.74822 (7)	0.43637 (4)	0.04498 (4)	0.04225 (16)	
F22	0.61743 (6)	0.60467 (3)	0.11394 (3)	0.03073 (12)	
F23	0.44244 (6)	0.55794 (3)	0.08514 (3)	0.03263 (12)	
F24	0.59871 (6)	0.54889 (3)	0.01780 (3)	0.03319 (12)	
F25	0.96494 (6)	0.46007 (4)	0.30957 (4)	0.03830 (14)	
F26	0.86681 (6)	0.53893 (4)	0.25256 (3)	0.03504 (13)	
F27	0.96813 (6)	0.56408 (4)	0.34683 (4)	0.04336 (17)	
F28	0.86092 (7)	0.42067 (3)	0.43543 (3)	0.03476 (13)	
F29	0.69549 (6)	0.47610 (3)	0.46362 (3)	0.03220 (12)	
F30	0.87415 (6)	0.52693 (4)	0.46509 (3)	0.03517 (13)	
O1	0.29692 (6)	0.34050 (3)	0.37637 (3)	0.02293 (10)	
O2	0.52465 (6)	0.34968 (3)	0.43109 (3)	0.02254 (10)	
O3	0.44219 (6)	0.20753 (3)	0.38991 (3)	0.02446 (11)	
O4	0.63661 (6)	0.25207 (3)	0.32627 (3)	0.02330 (11)	
O5	0.37785 (6)	0.26714 (3)	0.24941 (3)	0.02023 (10)	
H5	0.3322 (16)	0.2404 (9)	0.2369 (9)	0.049 (4)*	
O6	0.50145 (5)	0.37714 (3)	0.26817 (3)	0.01686 (9)	
O7	0.28867 (6)	0.39253 (3)	0.17783 (3)	0.02079 (10)	
O8	0.49285 (6)	0.31877 (3)	0.12510 (3)	0.02178 (10)	
O9	0.47985 (5)	0.52008 (3)	0.31503 (3)	0.01874 (9)	
O10	0.58483 (6)	0.49367 (3)	0.19124 (3)	0.02288 (11)	
O11	0.70824 (5)	0.45135 (3)	0.32217 (3)	0.01976 (10)	
C1	0.17918 (9)	0.34707 (5)	0.33840 (5)	0.02870 (16)	
H1A	0.1831 (14)	0.3858 (8)	0.3053 (8)	0.038 (4)*	
H1C	0.1646 (15)	0.3046 (8)	0.3152 (8)	0.040 (4)*	
H1B	0.1156 (14)	0.3533 (8)	0.3718 (8)	0.040 (4)*	
C2	0.32258 (10)	0.39816 (5)	0.42095 (5)	0.02961 (17)	
H2A	0.3473 (14)	0.4374 (8)	0.3930 (8)	0.037 (4)*	
H2B	0.2469 (14)	0.4092 (8)	0.4451 (8)	0.039 (4)*	
C3	0.42423 (10)	0.37570 (5)	0.47120 (5)	0.03070 (17)	
H3B	0.4521 (13)	0.4121 (7)	0.4995 (7)	0.031 (3)*	
H3A	0.3943 (14)	0.3386 (8)	0.5004 (8)	0.035 (4)*	
C4	0.62251 (11)	0.32156 (5)	0.47528 (5)	0.03130 (18)	
H4B	0.6448 (14)	0.3529 (8)	0.5115 (8)	0.037 (4)*	
H4C	0.6937 (16)	0.3137 (8)	0.4477 (9)	0.045 (4)*	
H4A	0.5926 (15)	0.2781 (9)	0.4935 (9)	0.045 (4)*	
C5	0.32458 (11)	0.18162 (5)	0.41272 (6)	0.0359 (2)	
H5C	0.2789 (14)	0.2185 (8)	0.4320 (8)	0.038 (4)*	
H5A	0.2754 (16)	0.1637 (9)	0.3727 (9)	0.048 (4)*	
H5B	0.3371 (17)	0.1489 (9)	0.4475 (10)	0.052 (5)*	
C6	0.51608 (9)	0.15551 (4)	0.35816 (5)	0.02861 (16)	
H6A	0.4813 (12)	0.1462 (7)	0.3124 (7)	0.027 (3)*	
H6B	0.5159 (13)	0.1139 (8)	0.3857 (8)	0.034 (4)*	
C7	0.64631 (9)	0.18360 (4)	0.35300 (5)	0.02821 (16)	
H7A	0.6873 (14)	0.1849 (8)	0.3989 (8)	0.035 (4)*	
H7B	0.6992 (13)	0.1562 (7)	0.3216 (7)	0.029 (3)*	
C8	0.75742 (9)	0.28065 (5)	0.31208 (6)	0.03016 (17)	
H8A	0.8135 (15)	0.2785 (8)	0.3543 (8)	0.043 (4)*	
H8C	0.7467 (14)	0.3266 (8)	0.2988 (8)	0.038 (4)*	
H8B	0.7958 (14)	0.2541 (8)	0.2782 (8)	0.039 (4)*	
C9	0.14136 (8)	0.46345 (5)	0.11889 (5)	0.02515 (14)	
C10	0.21298 (7)	0.39570 (4)	0.11929 (4)	0.02025 (12)	
H10	0.2557 (12)	0.3934 (7)	0.0767 (7)	0.024 (3)*	
C11	0.12361 (9)	0.33443 (5)	0.11643 (5)	0.02846 (16)	
C12	0.64542 (10)	0.23239 (5)	0.10666 (5)	0.03190 (18)	
C13	0.51129 (8)	0.25717 (4)	0.09276 (4)	0.02233 (13)	
H13	0.4584 (12)	0.2203 (6)	0.1078 (7)	0.022 (3)*	
C14	0.48145 (9)	0.26547 (5)	0.01520 (4)	0.02813 (16)	
C15	0.48497 (8)	0.64011 (4)	0.29406 (5)	0.02447 (14)	
C16	0.41558 (7)	0.57232 (4)	0.28234 (4)	0.01904 (12)	
H16	0.4087 (11)	0.5678 (6)	0.2321 (6)	0.021 (3)*	
C17	0.28263 (8)	0.57470 (4)	0.30916 (5)	0.02403 (14)	
C18	0.73865 (9)	0.46338 (5)	0.10836 (5)	0.02699 (15)	
C19	0.60311 (8)	0.48311 (4)	0.12248 (4)	0.02093 (12)	
H19	0.5527 (13)	0.4469 (7)	0.1023 (7)	0.030 (3)*	
C20	0.56621 (8)	0.54919 (4)	0.08447 (4)	0.02334 (14)	
C21	0.89578 (8)	0.51608 (5)	0.31614 (5)	0.02559 (15)	
C22	0.77568 (7)	0.50234 (4)	0.35500 (4)	0.01897 (12)	
H22	0.7350 (12)	0.5469 (7)	0.3583 (7)	0.025 (3)*	
C23	0.80330 (8)	0.48113 (4)	0.43008 (4)	0.02438 (14)	

### Atomic displacement parameters (Å^2^)

**Table:**

	*U*^11^	*U*^22^	*U*^33^	*U*^12^	*U*^13^	*U*^23^
Al1	0.01831 (9)	0.01458 (9)	0.01574 (9)	0.00019 (7)	−0.00074 (7)	−0.00034 (7)
Al2	0.01654 (9)	0.01461 (9)	0.01512 (9)	−0.00019 (7)	0.00018 (7)	0.00011 (7)
Mg1	0.02029 (11)	0.01498 (10)	0.01656 (11)	0.00142 (8)	0.00043 (9)	0.00067 (8)
F1	0.0274 (3)	0.0332 (3)	0.0347 (3)	0.0076 (2)	0.0061 (2)	−0.0026 (2)
F2	0.0327 (3)	0.0230 (3)	0.0664 (5)	−0.0001 (2)	0.0036 (3)	0.0121 (3)
F3	0.0431 (4)	0.0506 (4)	0.0329 (3)	0.0187 (3)	−0.0094 (3)	0.0102 (3)
F4	0.0331 (3)	0.0345 (3)	0.0642 (5)	−0.0082 (2)	0.0178 (3)	−0.0005 (3)
F5	0.0364 (3)	0.0228 (2)	0.0407 (3)	0.0001 (2)	−0.0032 (2)	−0.0059 (2)
F6	0.0488 (4)	0.0464 (4)	0.0577 (5)	0.0000 (3)	−0.0317 (4)	−0.0112 (3)
F7	0.0272 (3)	0.0464 (4)	0.0694 (5)	0.0010 (3)	−0.0019 (3)	−0.0088 (4)
F8	0.0619 (5)	0.0781 (6)	0.0311 (3)	0.0354 (5)	−0.0087 (3)	0.0025 (4)
F9	0.0512 (4)	0.0343 (3)	0.0523 (4)	0.0177 (3)	0.0092 (3)	−0.0090 (3)
F10	0.0406 (3)	0.0548 (4)	0.0280 (3)	0.0016 (3)	0.0094 (3)	0.0131 (3)
F11	0.0321 (3)	0.0574 (4)	0.0278 (3)	0.0024 (3)	−0.0062 (2)	−0.0015 (3)
F12	0.0884 (6)	0.0434 (4)	0.0309 (3)	0.0099 (4)	−0.0071 (4)	−0.0201 (3)
F13	0.0451 (3)	0.0271 (3)	0.0307 (3)	−0.0072 (2)	−0.0010 (2)	−0.0088 (2)
F14	0.0308 (3)	0.0285 (3)	0.0450 (3)	−0.0077 (2)	0.0078 (2)	−0.0005 (2)
F15	0.0503 (4)	0.0182 (2)	0.0465 (4)	0.0015 (2)	−0.0046 (3)	0.0093 (2)
F16	0.0319 (3)	0.0528 (4)	0.0313 (3)	−0.0010 (3)	0.0091 (2)	−0.0138 (3)
F17	0.0242 (2)	0.0264 (2)	0.0424 (3)	−0.00347 (19)	−0.0005 (2)	−0.0038 (2)
F18	0.0292 (3)	0.0297 (3)	0.0692 (5)	0.0119 (2)	−0.0024 (3)	0.0103 (3)
F19	0.0233 (2)	0.0422 (3)	0.0397 (3)	−0.0061 (2)	0.0044 (2)	−0.0007 (3)
F20	0.0380 (3)	0.0344 (3)	0.0378 (3)	0.0111 (2)	−0.0008 (3)	0.0007 (2)
F21	0.0451 (4)	0.0529 (4)	0.0293 (3)	0.0044 (3)	0.0098 (3)	−0.0140 (3)
F22	0.0386 (3)	0.0233 (2)	0.0297 (3)	−0.0088 (2)	−0.0080 (2)	0.00409 (19)
F23	0.0251 (3)	0.0375 (3)	0.0350 (3)	−0.0014 (2)	−0.0043 (2)	0.0082 (2)
F24	0.0394 (3)	0.0418 (3)	0.0183 (2)	−0.0107 (2)	0.0001 (2)	0.0072 (2)
F25	0.0263 (3)	0.0432 (3)	0.0460 (4)	0.0102 (2)	0.0106 (3)	−0.0001 (3)
F26	0.0377 (3)	0.0402 (3)	0.0276 (3)	−0.0050 (2)	0.0060 (2)	0.0064 (2)
F27	0.0316 (3)	0.0530 (4)	0.0457 (4)	−0.0215 (3)	0.0060 (3)	−0.0140 (3)
F28	0.0428 (3)	0.0315 (3)	0.0295 (3)	0.0118 (2)	−0.0062 (2)	0.0031 (2)
F29	0.0361 (3)	0.0365 (3)	0.0244 (2)	−0.0020 (2)	0.0077 (2)	−0.0015 (2)
F30	0.0378 (3)	0.0406 (3)	0.0265 (3)	−0.0049 (2)	−0.0085 (2)	−0.0095 (2)
O1	0.0240 (3)	0.0205 (2)	0.0244 (3)	0.00244 (19)	0.0033 (2)	−0.00119 (19)
O2	0.0286 (3)	0.0220 (2)	0.0170 (2)	0.0018 (2)	0.0000 (2)	−0.00050 (18)
O3	0.0308 (3)	0.0172 (2)	0.0256 (3)	0.0007 (2)	0.0030 (2)	0.00293 (19)
O4	0.0218 (2)	0.0196 (2)	0.0284 (3)	0.00416 (19)	−0.0010 (2)	−0.0006 (2)
O5	0.0246 (3)	0.0155 (2)	0.0205 (2)	−0.00338 (18)	−0.00220 (19)	0.00048 (17)
O6	0.0194 (2)	0.0147 (2)	0.0165 (2)	−0.00084 (16)	−0.00088 (17)	−0.00027 (15)
O7	0.0213 (2)	0.0201 (2)	0.0207 (2)	0.00311 (18)	−0.00353 (19)	−0.00046 (18)
O8	0.0271 (3)	0.0192 (2)	0.0192 (2)	0.00076 (19)	0.0030 (2)	−0.00330 (18)
O9	0.0221 (2)	0.0158 (2)	0.0182 (2)	0.00351 (17)	0.00021 (18)	0.00063 (16)
O10	0.0296 (3)	0.0233 (3)	0.0159 (2)	0.0006 (2)	0.0033 (2)	0.00277 (18)
O11	0.0188 (2)	0.0186 (2)	0.0217 (2)	0.00000 (17)	−0.00343 (18)	−0.00258 (18)
C1	0.0224 (4)	0.0315 (4)	0.0323 (4)	0.0038 (3)	0.0033 (3)	0.0013 (3)
C2	0.0323 (4)	0.0255 (4)	0.0314 (4)	0.0050 (3)	0.0057 (3)	−0.0073 (3)
C3	0.0364 (5)	0.0350 (4)	0.0210 (3)	0.0013 (3)	0.0057 (3)	−0.0065 (3)
C4	0.0396 (5)	0.0304 (4)	0.0233 (4)	0.0023 (3)	−0.0094 (3)	−0.0001 (3)
C5	0.0415 (5)	0.0237 (4)	0.0431 (5)	−0.0048 (3)	0.0132 (4)	0.0057 (4)
C6	0.0332 (4)	0.0168 (3)	0.0356 (4)	0.0042 (3)	−0.0025 (3)	−0.0003 (3)
C7	0.0295 (4)	0.0202 (3)	0.0346 (4)	0.0073 (3)	−0.0050 (3)	−0.0003 (3)
C8	0.0220 (4)	0.0286 (4)	0.0400 (5)	0.0028 (3)	0.0020 (3)	−0.0042 (3)
C9	0.0220 (3)	0.0260 (4)	0.0274 (4)	0.0044 (3)	−0.0003 (3)	0.0060 (3)
C10	0.0196 (3)	0.0227 (3)	0.0184 (3)	0.0023 (2)	−0.0001 (2)	0.0015 (2)
C11	0.0253 (4)	0.0268 (4)	0.0329 (4)	−0.0008 (3)	−0.0049 (3)	−0.0038 (3)
C12	0.0364 (5)	0.0312 (4)	0.0281 (4)	0.0113 (3)	−0.0002 (3)	−0.0035 (3)
C13	0.0287 (4)	0.0193 (3)	0.0191 (3)	0.0010 (2)	0.0021 (3)	−0.0019 (2)
C14	0.0338 (4)	0.0307 (4)	0.0199 (3)	0.0019 (3)	0.0010 (3)	−0.0048 (3)
C15	0.0294 (4)	0.0172 (3)	0.0267 (4)	−0.0003 (3)	−0.0001 (3)	0.0004 (2)
C16	0.0217 (3)	0.0159 (3)	0.0194 (3)	0.0017 (2)	−0.0008 (2)	−0.0003 (2)
C17	0.0230 (3)	0.0199 (3)	0.0290 (4)	0.0036 (2)	−0.0011 (3)	−0.0024 (3)
C18	0.0276 (4)	0.0304 (4)	0.0231 (3)	0.0008 (3)	0.0035 (3)	−0.0034 (3)
C19	0.0233 (3)	0.0223 (3)	0.0173 (3)	−0.0040 (2)	0.0014 (2)	0.0011 (2)
C20	0.0252 (3)	0.0260 (3)	0.0187 (3)	−0.0069 (3)	−0.0022 (3)	0.0039 (2)
C21	0.0210 (3)	0.0288 (4)	0.0270 (4)	−0.0030 (3)	0.0016 (3)	−0.0030 (3)
C22	0.0174 (3)	0.0193 (3)	0.0201 (3)	0.0004 (2)	−0.0006 (2)	−0.0022 (2)
C23	0.0268 (4)	0.0252 (3)	0.0210 (3)	0.0015 (3)	−0.0022 (3)	−0.0031 (3)

### Geometric parameters (Å, º)

**Table:**

Al1—Mg1	2.8074 (3)	O2—C4	1.4384 (11)
Al1—O5	1.7644 (6)	O3—C5	1.4370 (12)
Al1—O6	1.7456 (6)	O3—C6	1.4365 (11)
Al1—O7	1.7374 (6)	O4—C7	1.4367 (11)
Al1—O8	1.7425 (6)	O4—C8	1.4409 (11)
Al2—O6	1.7384 (6)	O5—H5	0.750 (18)
Al2—O9	1.7645 (6)	O7—C10	1.3713 (9)
Al2—O10	1.7307 (6)	O8—C13	1.3731 (9)
Al2—O11	1.7472 (6)	O9—C16	1.3731 (9)
Mg1—O1	2.1175 (7)	O10—C19	1.3644 (9)
Mg1—O2	2.1021 (6)	O11—C22	1.3731 (9)
Mg1—O3	2.1185 (6)	C1—H1A	0.993 (16)
Mg1—O4	2.0813 (6)	C1—H1C	0.953 (16)
Mg1—O5	2.0470 (6)	C1—H1B	0.960 (16)
Mg1—O6	2.0383 (6)	C2—H2A	0.979 (15)
F1—C9	1.3252 (11)	C2—H2B	0.970 (16)
F2—C9	1.3336 (11)	C2—C3	1.4998 (15)
F3—C9	1.3384 (11)	C3—H3B	0.940 (14)
F4—C11	1.3296 (12)	C3—H3A	0.978 (15)
F5—C11	1.3491 (11)	C4—H4B	0.954 (15)
F6—C11	1.3337 (11)	C4—H4C	0.955 (17)
F7—C12	1.3242 (14)	C4—H4A	0.977 (17)
F8—C12	1.3320 (12)	C5—H5C	0.953 (16)
F9—C12	1.3417 (12)	C5—H5A	0.985 (18)
F10—C14	1.3239 (12)	C5—H5B	0.934 (18)
F11—C14	1.3406 (12)	C6—H6A	0.965 (13)
F12—C14	1.3344 (11)	C6—H6B	0.973 (15)
F13—C15	1.3372 (11)	C6—C7	1.5023 (14)
F14—C15	1.3369 (11)	C7—H7A	0.977 (15)
F15—C15	1.3418 (10)	C7—H7B	0.997 (14)
F16—C17	1.3337 (11)	C8—H8A	0.997 (16)
F17—C17	1.3269 (10)	C8—H8C	0.940 (16)
F18—C17	1.3502 (10)	C8—H8B	0.940 (16)
F19—C18	1.3436 (11)	C9—C10	1.5289 (11)
F20—C18	1.3342 (11)	C10—H10	0.955 (13)
F21—C18	1.3401 (11)	C10—C11	1.5314 (12)
F22—C20	1.3341 (10)	C12—C13	1.5288 (13)
F23—C20	1.3342 (10)	C13—H13	0.966 (13)
F24—C20	1.3447 (10)	C13—C14	1.5309 (12)
F25—C21	1.3290 (11)	C15—C16	1.5313 (11)
F26—C21	1.3342 (11)	C16—H16	0.975 (12)
F27—C21	1.3418 (10)	C16—C17	1.5282 (12)
F28—C23	1.3347 (10)	C18—C19	1.5317 (12)
F29—C23	1.3430 (11)	C19—H19	0.964 (14)
F30—C23	1.3416 (10)	C19—C20	1.5308 (12)
O1—C1	1.4423 (11)	C21—C22	1.5308 (11)
O1—C2	1.4393 (11)	C22—H22	0.976 (13)
O2—C3	1.4362 (11)	C22—C23	1.5284 (11)
			
O5—Al1—Mg1	46.62 (2)	C6—C7—H7A	109.9 (9)
O6—Al1—Mg1	46.280 (19)	C6—C7—H7B	112.9 (8)
O6—Al1—O5	92.84 (3)	H7A—C7—H7B	108.6 (11)
O7—Al1—Mg1	121.25 (2)	O4—C8—H8A	110.3 (9)
O7—Al1—O5	115.19 (3)	O4—C8—H8C	108.7 (9)
O7—Al1—O6	109.19 (3)	O4—C8—H8B	109.4 (9)
O7—Al1—O8	108.90 (3)	H8A—C8—H8C	109.1 (13)
O8—Al1—Mg1	129.85 (2)	H8A—C8—H8B	106.3 (13)
O8—Al1—O5	112.28 (3)	H8C—C8—H8B	113.0 (13)
O8—Al1—O6	117.93 (3)	F1—C9—F2	106.94 (8)
O6—Al2—O9	114.57 (3)	F1—C9—F3	107.70 (7)
O6—Al2—O11	107.80 (3)	F1—C9—C10	113.08 (7)
O10—Al2—O6	111.99 (3)	F2—C9—F3	107.24 (8)
O10—Al2—O9	102.33 (3)	F2—C9—C10	110.02 (7)
O10—Al2—O11	115.11 (3)	F3—C9—C10	111.59 (8)
O11—Al2—O9	104.97 (3)	O7—C10—C9	108.99 (7)
O1—Mg1—Al1	95.31 (2)	O7—C10—H10	115.1 (8)
O1—Mg1—O3	91.48 (3)	O7—C10—C11	110.22 (7)
O2—Mg1—Al1	137.67 (2)	C9—C10—H10	106.8 (8)
O2—Mg1—O1	77.08 (3)	C9—C10—C11	111.41 (7)
O2—Mg1—O3	91.11 (3)	C11—C10—H10	104.2 (8)
O3—Mg1—Al1	131.01 (2)	F4—C11—F5	106.72 (8)
O4—Mg1—Al1	99.60 (2)	F4—C11—F6	107.79 (9)
O4—Mg1—O1	164.77 (3)	F4—C11—C10	113.43 (8)
O4—Mg1—O2	93.74 (3)	F5—C11—C10	108.94 (7)
O4—Mg1—O3	76.40 (3)	F6—C11—F5	106.86 (8)
O5—Mg1—Al1	38.791 (17)	F6—C11—C10	112.73 (8)
O5—Mg1—O1	91.83 (3)	F7—C12—F8	108.82 (10)
O5—Mg1—O2	168.38 (3)	F7—C12—F9	106.99 (9)
O5—Mg1—O3	92.66 (3)	F7—C12—C13	113.23 (8)
O5—Mg1—O4	97.83 (3)	F8—C12—F9	106.27 (9)
O6—Mg1—Al1	38.241 (16)	F8—C12—C13	108.92 (8)
O6—Mg1—O1	98.71 (3)	F9—C12—C13	112.33 (9)
O6—Mg1—O2	101.00 (2)	O8—C13—C12	110.17 (7)
O6—Mg1—O3	165.62 (3)	O8—C13—H13	115.0 (7)
O6—Mg1—O4	94.94 (3)	O8—C13—C14	108.92 (7)
O6—Mg1—O5	76.99 (2)	C12—C13—H13	105.4 (8)
C1—O1—Mg1	126.61 (5)	C12—C13—C14	111.77 (7)
C2—O1—Mg1	109.81 (5)	C14—C13—H13	105.6 (8)
C2—O1—C1	112.43 (7)	F10—C14—F11	106.54 (8)
C3—O2—Mg1	114.36 (6)	F10—C14—F12	107.70 (8)
C3—O2—C4	110.90 (7)	F10—C14—C13	112.93 (8)
C4—O2—Mg1	121.88 (5)	F11—C14—C13	110.17 (7)
C5—O3—Mg1	124.96 (6)	F12—C14—F11	106.74 (8)
C6—O3—Mg1	109.69 (5)	F12—C14—C13	112.41 (8)
C6—O3—C5	112.30 (7)	F13—C15—F15	107.31 (7)
C7—O4—Mg1	116.74 (5)	F13—C15—C16	113.31 (7)
C7—O4—C8	111.98 (7)	F14—C15—F13	106.81 (8)
C8—O4—Mg1	129.09 (5)	F14—C15—F15	107.11 (7)
Al1—O5—Mg1	94.59 (3)	F14—C15—C16	109.92 (7)
Al1—O5—H5	122.8 (13)	F15—C15—C16	112.06 (7)
Mg1—O5—H5	141.3 (13)	O9—C16—C15	110.00 (6)
Al1—O6—Mg1	95.48 (3)	O9—C16—H16	114.3 (7)
Al2—O6—Al1	128.72 (3)	O9—C16—C17	108.89 (6)
Al2—O6—Mg1	135.07 (3)	C15—C16—H16	104.3 (7)
C10—O7—Al1	131.50 (5)	C17—C16—C15	112.01 (6)
C13—O8—Al1	131.68 (5)	C17—C16—H16	107.4 (7)
C16—O9—Al2	124.61 (5)	F16—C17—F18	107.16 (7)
C19—O10—Al2	147.43 (6)	F16—C17—C16	113.48 (7)
C22—O11—Al2	128.68 (5)	F17—C17—F16	107.00 (8)
O1—C1—H1A	110.0 (9)	F17—C17—F18	106.62 (7)
O1—C1—H1C	106.8 (10)	F17—C17—C16	110.10 (7)
O1—C1—H1B	107.1 (9)	F18—C17—C16	112.11 (7)
H1A—C1—H1C	111.6 (13)	F19—C18—C19	112.73 (7)
H1A—C1—H1B	112.5 (13)	F20—C18—F19	107.59 (8)
H1C—C1—H1B	108.6 (13)	F20—C18—F21	107.68 (8)
O1—C2—H2A	109.4 (9)	F20—C18—C19	110.33 (7)
O1—C2—H2B	108.4 (9)	F21—C18—F19	107.10 (8)
O1—C2—C3	106.19 (7)	F21—C18—C19	111.19 (8)
H2A—C2—H2B	109.6 (13)	O10—C19—C18	112.11 (7)
C3—C2—H2A	112.6 (9)	O10—C19—H19	114.2 (8)
C3—C2—H2B	110.6 (9)	O10—C19—C20	107.27 (7)
O2—C3—C2	107.02 (7)	C18—C19—H19	105.3 (8)
O2—C3—H3B	110.7 (9)	C20—C19—C18	110.97 (7)
O2—C3—H3A	108.2 (9)	C20—C19—H19	107.0 (8)
C2—C3—H3B	111.4 (9)	F22—C20—F23	106.59 (8)
C2—C3—H3A	110.2 (9)	F22—C20—F24	107.31 (7)
H3B—C3—H3A	109.3 (12)	F22—C20—C19	112.61 (7)
O2—C4—H4B	110.5 (9)	F23—C20—F24	107.19 (7)
O2—C4—H4C	107.9 (10)	F23—C20—C19	110.13 (7)
O2—C4—H4A	107.8 (10)	F24—C20—C19	112.68 (7)
H4B—C4—H4C	109.1 (13)	F25—C21—F26	107.54 (8)
H4B—C4—H4A	111.8 (13)	F25—C21—F27	107.66 (8)
H4C—C4—H4A	109.7 (14)	F25—C21—C22	112.32 (7)
O3—C5—H5C	108.5 (9)	F26—C21—F27	106.68 (8)
O3—C5—H5A	109.6 (10)	F26—C21—C22	109.65 (7)
O3—C5—H5B	110.8 (11)	F27—C21—C22	112.72 (7)
H5C—C5—H5A	108.0 (13)	O11—C22—C21	109.68 (6)
H5C—C5—H5B	107.4 (14)	O11—C22—H22	116.7 (8)
H5A—C5—H5B	112.4 (15)	O11—C22—C23	108.94 (6)
O3—C6—H6A	108.8 (8)	C21—C22—H22	104.9 (8)
O3—C6—H6B	110.4 (9)	C23—C22—C21	111.90 (7)
O3—C6—C7	107.01 (7)	C23—C22—H22	104.7 (8)
H6A—C6—H6B	109.7 (12)	F28—C23—F29	107.37 (8)
C7—C6—H6A	109.8 (8)	F28—C23—F30	107.43 (7)
C7—C6—H6B	111.1 (9)	F28—C23—C22	112.86 (7)
O4—C7—C6	107.91 (7)	F29—C23—C22	109.54 (7)
O4—C7—H7A	109.1 (9)	F30—C23—F29	106.55 (7)
O4—C7—H7B	108.5 (8)	F30—C23—C22	112.76 (7)
			
Al1—O7—C10—C9	−164.38 (6)	O6—Al2—O10—C19	−31.10 (12)
Al1—O7—C10—C11	73.05 (9)	O6—Al2—O11—C22	−160.88 (6)
Al1—O8—C13—C12	−104.13 (8)	O7—Al1—O5—Mg1	−110.14 (3)
Al1—O8—C13—C14	132.95 (7)	O7—Al1—O6—Al2	−56.01 (5)
Al2—O9—C16—C15	−105.20 (7)	O7—Al1—O6—Mg1	115.29 (3)
Al2—O9—C16—C17	131.71 (6)	O7—Al1—O8—C13	−116.23 (7)
Al2—O10—C19—C18	−73.76 (12)	O7—C10—C11—F4	61.25 (10)
Al2—O10—C19—C20	164.18 (8)	O7—C10—C11—F5	−57.46 (9)
Al2—O11—C22—C21	−107.26 (7)	O7—C10—C11—F6	−175.90 (8)
Al2—O11—C22—C23	129.95 (6)	O8—Al1—O5—Mg1	124.45 (3)
Mg1—Al1—O6—Al2	−171.30 (6)	O8—Al1—O6—Al2	68.93 (5)
Mg1—Al1—O7—C10	−148.45 (6)	O8—Al1—O6—Mg1	−119.77 (3)
Mg1—Al1—O8—C13	63.88 (8)	O8—Al1—O7—C10	31.66 (8)
Mg1—O1—C2—C3	−49.83 (8)	O8—C13—C14—F10	62.80 (10)
Mg1—O2—C3—C2	−32.68 (9)	O8—C13—C14—F11	−56.19 (10)
Mg1—O3—C6—C7	50.27 (8)	O8—C13—C14—F12	−175.09 (8)
Mg1—O4—C7—C6	21.82 (9)	O9—Al2—O6—Al1	97.72 (4)
F1—C9—C10—O7	−62.37 (9)	O9—Al2—O6—Mg1	−69.97 (5)
F1—C9—C10—C11	59.47 (10)	O9—Al2—O10—C19	−154.29 (10)
F2—C9—C10—O7	57.12 (9)	O9—Al2—O11—C22	−38.33 (7)
F2—C9—C10—C11	178.96 (8)	O9—C16—C17—F16	57.58 (9)
F3—C9—C10—O7	176.03 (7)	O9—C16—C17—F17	−62.31 (9)
F3—C9—C10—C11	−62.13 (10)	O9—C16—C17—F18	179.17 (7)
F7—C12—C13—O8	−53.87 (10)	O10—Al2—O6—Al1	−18.24 (5)
F7—C12—C13—C14	67.37 (10)	O10—Al2—O6—Mg1	174.06 (4)
F8—C12—C13—O8	67.34 (11)	O10—Al2—O9—C16	14.74 (7)
F8—C12—C13—C14	−171.42 (9)	O10—Al2—O11—C22	73.34 (7)
F9—C12—C13—O8	−175.20 (8)	O10—C19—C20—F22	48.57 (9)
F9—C12—C13—C14	−53.97 (11)	O10—C19—C20—F23	−70.24 (8)
F13—C15—C16—O9	−63.10 (9)	O10—C19—C20—F24	170.13 (7)
F13—C15—C16—C17	58.14 (9)	O11—Al2—O6—Al1	−145.85 (4)
F14—C15—C16—O9	56.31 (9)	O11—Al2—O6—Mg1	46.46 (5)
F14—C15—C16—C17	177.55 (7)	O11—Al2—O9—C16	135.28 (6)
F15—C15—C16—O9	175.29 (7)	O11—Al2—O10—C19	92.49 (11)
F15—C15—C16—C17	−63.47 (9)	O11—C22—C23—F28	55.14 (9)
F19—C18—C19—O10	−75.70 (9)	O11—C22—C23—F29	−64.43 (8)
F19—C18—C19—C20	44.23 (10)	O11—C22—C23—F30	177.10 (7)
F20—C18—C19—O10	44.60 (10)	C1—O1—C2—C3	163.61 (8)
F20—C18—C19—C20	164.53 (7)	C4—O2—C3—C2	−175.18 (8)
F21—C18—C19—O10	164.01 (7)	C5—O3—C6—C7	−166.16 (8)
F21—C18—C19—C20	−76.06 (9)	C8—O4—C7—C6	−173.46 (8)
F25—C21—C22—O11	−56.24 (9)	C9—C10—C11—F4	−59.88 (10)
F25—C21—C22—C23	64.78 (9)	C9—C10—C11—F5	−178.59 (7)
F26—C21—C22—O11	63.25 (9)	C9—C10—C11—F6	62.97 (10)
F26—C21—C22—C23	−175.73 (7)	C12—C13—C14—F10	−59.15 (10)
F27—C21—C22—O11	−178.07 (7)	C12—C13—C14—F11	−178.15 (8)
F27—C21—C22—C23	−57.05 (10)	C12—C13—C14—F12	62.95 (11)
O1—C2—C3—O2	53.21 (10)	C15—C16—C17—F16	−64.29 (9)
O3—C6—C7—O4	−46.18 (10)	C15—C16—C17—F17	175.82 (7)
O5—Al1—O6—Al2	−173.95 (4)	C15—C16—C17—F18	57.30 (9)
O5—Al1—O6—Mg1	−2.65 (3)	C18—C19—C20—F22	−74.20 (9)
O5—Al1—O7—C10	−95.49 (7)	C18—C19—C20—F23	166.98 (7)
O5—Al1—O8—C13	12.55 (8)	C18—C19—C20—F24	47.35 (9)
O6—Al1—O5—Mg1	2.63 (3)	C21—C22—C23—F28	−66.31 (9)
O6—Al1—O7—C10	161.70 (7)	C21—C22—C23—F29	174.12 (7)
O6—Al1—O8—C13	118.68 (7)	C21—C22—C23—F30	55.65 (9)
O6—Al2—O9—C16	−106.68 (6)		

### Hydrogen-bond geometry (Å, º)

**Table:**

*D*—H···*A*	*D*—H	H···*A*	*D*···*A*	*D*—H···*A*
O5—H5···F18^i^	0.750 (18)	2.315 (18)	2.9816 (8)	148.7 (17)
C2—H2*B*···F30^ii^	0.970 (16)	2.527 (16)	3.4204 (11)	153.1 (12)
C4—H4*A*···F6^iii^	0.977 (17)	2.539 (17)	3.5007 (13)	168.1 (13)
C7—H7*B*···F22^iv^	0.997 (14)	2.500 (14)	3.0101 (10)	111.3 (10)
C8—H8*C*···O11	0.940 (16)	2.515 (16)	3.3831 (11)	153.7 (13)
C19—H19···O8	0.964 (14)	2.625 (14)	3.4214 (10)	140.2 (11)

Symmetry codes: (i) −*x*+1/2, *y*−1/2, −*z*+1/2; (ii) −*x*+1, −*y*+1, −*z*+1; (iii) *x*+1/2, −*y*+1/2, *z*+1/2; (iv) −*x*+3/2, *y*−1/2, −*z*+1/2.
